# Strategies and hurdles using DNA vaccines to fish

**DOI:** 10.1186/1297-9716-45-21

**Published:** 2014-02-19

**Authors:** Linn B Hølvold, Anne I Myhr, Roy A Dalmo

**Affiliations:** 1 UiT - The Arctic University of Norway, Faculty of Biosciences, Fisheries & Economics, Norwegian College of Fishery Science 9037 Tromsø, Norway; 2 Genøk – Centre for Biosafety, The Science Park, Postbox 6418, 9294 Tromsø Norway

## Abstract

DNA vaccinations against fish viral diseases as IHNV at commercial level in Canada against VHSV at experimental level are both success stories. DNA vaccination strategies against many other viral diseases have, however, not yet yielded sufficient results in terms of protection. There is an obvious need to combat many other viral diseases within aquaculture where inactivated vaccines fail. There are many explanations to why DNA vaccine strategies against other viral diseases fail to induce protective immune responses in fish. These obstacles include: 1) too low immunogenicity of the transgene, 2) too low expression of the transgene that is supposed to induce protection, 3) suboptimal immune responses, and 4) too high degradation rate of the delivered plasmid DNA. There are also uncertainties with regard distribution and degradation of DNA vaccines that may have implications for safety and regulatory requirements that need to be clarified. By combining plasmid DNA with different kind of adjuvants one can increase the immunogenicity of the transgene antigen – and perhaps increase the vaccine efficacy. By using molecular adjuvants with or without in combination with targeting assemblies one may expect different responses compared with naked DNA. This includes targeting of DNA vaccines to antigen presenting cells as a central factor in improving their potencies and efficacies by means of encapsulating the DNA vaccine in certain carriers systems that may increase transgene and MHC expression. This review will focus on DNA vaccine delivery, by the use of biodegradable PLGA particles as vehicles for plasmid DNA mainly in fish.

## Table of contents

1. Introduction

2. DNA vaccines and vaccination

3. Immune responses to DNA vaccination – mainly in mice and birds

4. DNA vaccination against novirhabdoviruses

5. Recent DNA vaccination laboratory trials

6. Administration and distribution of DNA vaccines

6.1. Intramuscular injection

6.2. Other routes of delivery

7. Uptake of plasmid DNA in fish and mammalian species

8. Factors influencing transfection and transgene expression

9. Advantages, disadvantages and challenges of DNA vaccines

10. Application of molecular adjuvants to increase transgene immunogenicity

11. PLGA particles as carrier systems for DNA vaccines – focus on fish

12. Issues of making DNA encapsulated PLGA particles

13. Transgene expression and immune responses by PLGA-encapsulated pDNA

14. Other particles in vaccine delivery to fish

15. Current challenges in the use of PLGA particles as carriers/adjuvants

16. Concerns regarding PLGA nano- and micro-particles

17. Safety and regulatory aspects by DNA vaccines

18. Conclusions

19. Competing interests

20. Authors’ contributions

21. Acknowledgements

22. References

## 1. Introduction

There are quite a limited number of DNA vaccine strategies that have been successful in giving significant protection against fish diseases. The excellent exceptions are DNA vaccination against infectious hematopoietic necrosis virus (IHNV) at commercial level and against viral hemorrhagic septicemia virus (VHSV) at experimental/small scale level [1]. A promising strategy to increase the DNA vaccine efficacy, against other viral diseases, is to target the construct and/transgene to antigen presenting cells. Wang et al. [2] have presented an overview on how DNA vaccines can be targeted to antigen presenting cells (APC) and dendritic cells (DC) using molecular assemblies with the resulting immune responses. Another strategy is to encapsulate the DNA vaccine in certain carriers with the intention to increase transgene and MHC expression. There are many particulate carriers that have been explored to present and target DNA vaccines to desired cells and tissues, one of them being poly (D,L-lactic-co-glycolic)-acid (PLGA) particles.

## 2. DNA vaccines and vaccination

The definition of DNA vaccination as provided by The Norwegian Biotechnology Advisory Board [3] is “the intentional transfer of genetic material (DNA or RNA) to somatic cells for the purpose of influencing the immune system”. This sets it slightly apart from gene therapy, which in the same report is referred to as an introduction of novel gene(s) for purposes other than influencing the immune system. The mechanism of a DNA vaccine can in many ways be likened to that of a virus, as it requires the same cellular machinery in order to replicate and since it also triggers immune responses normally seen with viral infections [4]. Unlike conventional viral vaccines based on subunits or killed virus, a DNA vaccine may conserve the structure and hence also antigenicity of a transgenic antigen/protein [5].

## 3. Immune responses to DNA vaccination – mainly in mice and birds

A vital attribute of DNA vaccines is the ability to induce all three arms of adaptive immunity, namely; helper T-cells, cytotoxic T cells (CTLs) and antibodies, although they were initially investigated in the attempt to find ways of delivering antigen to major histocompatibility complex (MHC) class I and induce T helper 1 (Th1) responses [6,7]. Professional APCs are the cells that primarily contribute to the immune responses to DNA vaccination. Direct transfection of DCs provides the most efficient priming of naïve CTLs, and is perhaps the major mechanism for priming of these cells [8]. APCs are also able to take up exogenous antigens and process them for presentation by either MHC class II, or MHC class I following transfer of the antigen to the cytosol (cross-priming) [9]. These responses are vital in the cellular immune response following DNA vaccination. The expression of reporter genes is not only higher in fish, but also seems to have a longer duration. Transgene expression has been detected at the injection site as long as two years after injection of glass catfish (*Kryptopterus bicirrhus*) [10], and Tonheim et al. detected both supercoiled (sc) DNA and luciferase expression at the injection site 535 days after intramuscular injection of Atlantic salmon (*Salmo salar* L.) with pDNA [11].

The immunogenicity of DNA vaccines stems not only from the expression of the GOI, but also from properties of the plasmid vectors themselves. The ability of foreign nucleic acids to induce interferons (IFNs) in mouse fibroblasts was discovered by two independent research groups as early as in 1963 [12]. The most studied pDNA property in terms of possible adjuvant effects is CpG motifs, regions in the DNA where a cytosine nucleotide occurs next to a guanine nucleotide and the two are linked by a phosphodiester bond. In vertebrates these sequences are highly methylated, whereas they show a much lower methylation frequency in viral and bacterial DNA (such as pDNA). These CpG motifs can act as pathogen-associated molecular pattern molecules (PAMPs) and are recognized as danger signals by the vertebrate immune system, resulting in a release of cytokines, macrophage activation, a differentiation of Th1 effector cells as well as B-cell proliferation and secretion of antibodies [13]. Plasmid DNA containing optimized CpG content may be likely used in DNA vaccine strategies.

## 4. DNA vaccination against novirhabdoviruses

The first DNA vaccination of fish took place in 1996, when Anderson et al. immunized rainbow trout (*Onchorhyncus mykiss*) against IHNV [14]. Since then several trials have been performed for a wide variety of fish species and pathogens and in 2005 a vaccine against IHNV infection in salmonids (Apex-IHN®, Novartis Animal Health) was also one of the first DNA vaccine ever to be cleared for marketing (by the Canadian Food Inspection Agency). In 1999 the injection of Atlantic salmon with pCMV4-G (plasmid-encoded glycoprotein) from a rainbow trout IHNV isolate induced significant protection against challenges with IHNV, even though the salmon were much larger than the rainbow trout in previous challenge studies [15]. DNA vaccination of fish has been shown to induce both innate and adaptive immune responses similar to what is seen in mammalian species, and seems especially efficient against novirhabdoviruses (like VHSV and IHNV). These are simple RNA viruses with either five or six genes and a single viral surface protein (glycoprotein, or G protein) that acts as the protective antigen [16].

In rainbow trout an immunization against VHSV enables the induction of cell-mediated immune responses encompassing both CTLs and natural killer (NK) cells and has also been shown to significantly reduce the replication of virus during challenge [17]. Interestingly, when Cuesta et al. looked at the innate and adaptive responses in vaccinated and control fish after challenge, they found the highest increase in vaccinated fish to be that of innate immune responses [18]. Lorenzen et al. [19] have also demonstrated the importance of innate responses in early antiviral defense, wherein rainbow trout were subjected to VHSV challenge following vaccination with pIHN-G (plasmid-encoded IHNV glycoprotein). Whereas protection at late stages of the challenge could only be conferred by previous immunization with pVHS-G, the two vaccines induced similar levels of immune responses and protection during the first week following challenge.

In conclusion, anti novirhabdovirus response governed by DNA vaccines relies much on the immunogenicity of the G-protein resulting in long-lasting protection mediated by cellular and humoral responses. In addition, there is an innate and specific response following DNA vaccination that may be protective in a short-term perspective.

Other RNA viruses, or the larger DNA viruses, often offer more difficulty in identifying a protective antigen, although viral surface protein genes are almost always chosen for DNA vaccines [2]. Some pathogens, such as infectious pancreatic necrosis virus (IPNV), primarily cause disease in fry. Whereas vaccination by injection is highly impractical at this stage [20], the vaccination of post-smolts has been shown to induce protection upon challenge [21]. Good protection did, however, require the use of plasmids encoding all the large poly-proteins of the IPNV.

## 5. Recent DNA vaccination laboratory trials

DNA vaccines and their effects against several viral and bacterial diseases in fish have been reviewed by Tonheim et al*.*[22], Kurath [23], Redding and Weiner [24], and Gomez-Casado et al. [25]. Since these reviews were published, several new results using DNA vaccine strategies have been reported (Table 1).

**Table 1:** Experimental DNA vaccines and their protection in fish following experimental infection

**Pathogen**	**Gene inserted**	**Host**	**Administration route/adjuvant**	**Protection**	**References**
IHNV	IHNV-G plus suicidal gene	Rainbow trout	I.m/none	Yes	[26]
IHNV	IHNV-G; different genogroups	Rainbow trout	I.m/none	Yes; cross-protection	[27]
IHNV	IHNV-G	Rainbow trout	Oral/PLGA	No	[28]
VHSV	*E. tarda* as delivery vehicle of the vaccine	Olive/Japanese flounder (*Paralichthys olivaceus*)	I.m	Yes	[29]
IPNV	VP2		Oral/alginate	Yes	[30]
IPNV	VP2; Segment A of TA strain	Atlantic salmon	I.m	No	[31]
SAV	E1 and E2	Atlantic salmon	I.m	No	[32]
Megalocytivirus	86-residue VP	Turbot (*Scophthalmus maximus*)	I.m	Yes	[33]
*Edwardsiella tarda*	Eta6-FliC chimeric protein	Japanese flounder (*Paralichthys olivaceus*)	I.m	Yes	[34]
*E. tarda*	D15-like surface antigen	Japanese flounder	I.m	Yes	[35]
*E. tarda*	Eta2	Japanese flounder	i.m	Yes	[36]
*Streptococcus iniae*	sagF, sagG and sagI	Japanese flounder	I.m	Yes	[37]
*S. iniae*	Sia10 delivered by *E. tarda*	Japanese flounder	Oral/alginate, immersion- boosted	Yes	[38]
*Vibrio harveyi*	FlaA	Yellow grouper *(Epinephelus awoara)*	I.m	Yes	[39]
*V. harveyi*	DegQ or/and Vhp1	Japanese flounder	I.m	Yes	[40]
*V. alginolyticus*	FlaA	Red snapper (*Lutjanus campechanus*)	I.m	Yes	[41]
*S. iniae* and *V. anguillarum*	Sia10 and/or OmpU	Turbot	I.m	Yes, cross-protection	[37]
*Flavobacterium psychrophilum*	Hsp60, hsp70	Rainbow trout	I.m	No	[42]
*Cryptocaryon irritans*	iAg	Orange spotted grouper (*E. coioides)*	I.m	Yes	[43]
*Cryptobia salmocitica*	Metalloprotease	Atlantic salmon and rainbow trout	I.m	Partly	[44]
*Ichthyophthrius multifiliis*	Immunobilization antigens and cystein protease	Rainbow trout	I.m, gene gun and air pressure	No	[45]

Apparently, DNA vaccination may also confer protection against bacteria and parasites – but not against all. Bacteria and parasites may express and harbor numerous different antigens dependent on their life cycle, this make a DNA vaccination strategy more complicated. There are developed “traditional” oil adjuvanted vaccines against a number of bacterial diseases, where the strategies may be quite efficient preventing infection and disease. This is in contrast to some parasitic infections/attachment such as sea lice - where no immune prophylaxis exists at present at an industrial scale.

## 6. Administration and distribution of DNA vaccines

### 6.1. Intramuscular injection

Intramuscular injection is widely applied for pDNA delivery in fish and generally results in strong expressions of transgene at the injection site [22]. Studies in mice have found the dispersion of pDNA immediately following intramuscular injection to take place primarily between the muscle body and epimysium (connective tissue that enclosing the entire muscle). Myocytes and mononuclear cells take up pDNA after administration [22], but despite a rapid initiation of uptake the subsequent uptake is slow and cells along the muscle fibers have been shown to be transfected over a period of hours following injection. With very small fish this initial dispersion of a vaccine might be enough to ensure the perfusion of intact pDNA to more distant tissues, while in large fish the injected volume will mainly rest along the needle trajectory [22]. The transportation of pDNA to and from blood to other tissues has been reported for various fish species [22]. Plasmids have been recovered from sites such as liver, spleen, head-kidney, heart and intestine for some time after injection, but mainly persist at the site of injection. Degradation of the pDNA starts within five minutes following injection of mice, with as much as 95-99% of the initial pDNA amount degraded within 90 min [22]. The rate of degradation in the tissue of cold-water fish remains to be determined. The extent of histopathological changes at the injection site following intramuscular DNA delivery in fish appear to increase with an increase in vaccination dose [46], but vaccination will generally induce only moderate local tissue damage in form of degeneration of myocytes, hemorrhages and a transient influx of inflammatory cells [20].

### 6.2. Other routes of delivery

Other routes of pDNA administration that have been investigated in fish are intravenous, intraperitoneal, oral delivery, and particle bombardment [1,21]. Accumulation of naked pDNA took place primarily in the heart, kidney and liver following intravenous administration [22], whereas oral delivery resulted in a recovery of DNA fragments from the pyloric region, kidney, spleen and liver – assessed by revers-transcription polymerase chain reaction (RT-PCR) [28].

## 7. Uptake of plasmid DNA in fish and mammalian species

A wide variety of mammalian cell types has been shown to take up pDNA – reviewed by Budker et al. [47], but so far uptake in fish has only been reported for myocytes, head kidney macrophages and endocardial endothelial cells (EECs) [22]. The exact mechanisms by which myocytes take up pDNA remains to be determined, but several suggestions have been made [48]. It was previously theorized that direct injections caused temporary membrane disruptions and/or pores that allowed for the entry of pDNA [5], but studies have shown that such disruptions in fact work to abolish transfection [12].

Several PPRs have been shown to bind nucleic acids including plasmid DNA, reviewed by Desmet et al. [49]. Of the number of cell-surface receptors investigated in terms of DNA binding and uptake, scavenger receptors (SRs) in particular have been a subject of interest. These receptors comprise a broad family of membrane proteins capable of binding a wide range of anionic ligands and are present on several different cell types [50]. The uptake of pDNA by SRs has been demonstrated in Atlantic cod (*Gadus morhua*) atrial EECs [51], but although the SRs may bind DNA they appear not to be essential for the immunostimulatory activity of CpG DNA [52]. This indicates that SRs activity may be essential for the transgene expression – as they may bind and facilitate degradation of high amount of plasmid DNA before expression occurs.

## 8. Factors influencing transfection and transgene expression

Nucleic acids have poor intrinsic transfection efficiency due to their large size and negative charge [53]. The processes of gene transcription and translation are carried out by the cell’s own machinery, and may be influenced by a variety of factors such as pDNA vector design, pDNA concentrations, administration volumes, age and size of the fish, water temperatures and method as well as route of administration.

It has been well documented that the DNA topoform has a strong influence on the efficiency of transfection [54]. Supercoiled DNA is frequently reported as the most potent topoform followed by open circular (oc) forms, whereas linearization of the DNA has been shown to nearly abolish the expression of transgene and linearized pDNA. The choice of promoter also has a profound influence on the level of transgene expression [55] and the cytomegalovirus (CMV) promoter has often been reported as the most effective. Because of this, the CMV promoter is the most commonly applied in gene transfection studies and its potency has been demonstrated also in fish [5]. The use of an intron and poly-adenylation (termination) signals further improves expression [55].

Despite the inherent adjuvant effects of DNA vaccines, it has been shown that immune responses triggered by DNA vaccination may in fact limit the duration of transgene expression [55]. The lack of anti-Luc antibodies means that both levels and duration of expression are commonly higher in studies applying luciferase compared to more immunogenic antigens, and immune responses have only been observed with the application of large pDNA doses and potent adjuvants such as Freund´s complete adjuvant (FCA) [56]. A co-administration of vectors encoding Luc and immunogenic proteins (β-gal, G protein) decreases both the strength and duration of luciferase expression, as the initiated antigen specific cytotoxic responses work to eliminate transfected cells expressing the transgenic protein [56]. The stimulation of PRRs may also induce responses that can be detrimental to transgene expression. The hallmark cytokines of the inflammatory response, TNF-α and IL-1β, have both been shown to inhibit transgene expression in vitro and in vivo [57], as have the Th1 associated cytokines IFN-γ and IFN-α [58]. Levels of inhibition have in fact been shown to correlate with the levels of CpG-induced cytokines, and synergistic effects were also observed. The inhibitory effect takes place at the mRNA (post-transcriptional) level, hence not causing vector degradation, inhibition of total cellular protein synthesis or elimination of infected/transfected cells [58].

Whereas intramuscular injection is widely acknowledged as the superior method of administration to achieve high levels of transgene expression [1], the result still depends on factors such as dose, volume and fish size and age [59]. Transgene expression has been found to be higher in young and growing fish [5], and small fish sizes appear to favor not only the distribution of pDNA throughout tissue but the distribution of transgene expression as well [5]. For example expression of luciferase in thymus, gills, spleen and kidney has been reported for small fish (< 5 g), although the highest expression is consistently detected at the site of injection in myocytes, infiltrating cells and epithelial cells lining small capillaries [20,60].

The first reporter gene study in fish indicated the existence of a maximum above where there would be no further increase in expression [60]. This observation is supported by later findings in zebra fish (*Danio rerio*) and rainbow trout [5], and there are indications that excessive DNA concentrations may actually reduce transgene expression [21]. The injection of large volumes might contribute to a spatial distribution by creating temporary gaps between fibers [61], which appears to induce higher expression as well as reduce the variations commonly observed in in vivo transfection studies [5]. A pre-injection of an isotonic solution created the same effect, and lead to both higher and more equal transgene expression among individuals after DNA injection [61].

## 9. Advantages, disadvantages and challenges of DNA vaccines

The potency of DNA vaccines for inducing the different branches of both innate and adaptive immunity has already been described. DNA vaccines also show high efficiency when given at early life stages [62], and provide the benefit of inducing protective immunity over a wide span of temperatures [63].

The advantages of DNA vaccination still stretch beyond merely the immunological capacities. Looking at the concept from a manufacturer or/and investor’s standpoint, DNA vaccines are relatively inexpensive and easy to produce. The processes required for production are identical for all DNA vaccines, and the ease of cloning also enables rapid modifications in a way that is generally not obtainable with conventional vaccine preparations [60].

Potential side effects include e.g. risk of autoimmunity, immune tolerance against the expressed antigen, too high CTL response resulting in myositis, chromosomal integration, injection site inflammation and tissue destruction [1,64].

## 10. Application of molecular adjuvants to increase transgene immunogenicity

To increase immunogenicity of a given DNA vaccine one may consider optimization by including co-injected adjuvant, either being traditional aluminum salts, polysaccharides (e.g. zymosan, glucans, chitosan), different liposomes, synthetic polymers and TLR agonists. Moreover, plasmid encoded cytokine adjuvants may also be one approach to increase the immunogenicity. IL-2, IFN-γ, IL-12, GM-CSF and IL-15 have been shown to modulate immune responses when co-encoded by the DNA vaccine [65]. One may also apply the concept of immune modulating effects chemokines, transcription factors and/or co-stimulatory factors assembled into the plasmid vectors. Using fish models, only one report on the use of cytokine adjuvants encoded by the same plasmid as the expression plasmid has been published. Caipang et al. used Japanese flounder interferon regulatory factor-1 (IRF-1) cloned into a plasmid DNA vector containing the major capsid protein gene of sea bream iridovirus. The antibody levels of fish injected with this vaccine were not significant higher with the control plasmid without IRF-1 gene [66]. Unfortunately, no experimental virus challenge was carried out to monitor vaccine efficacy. To increase vaccine potency and efficacy of poor performing DNA vaccines one should explore strategies such as inclusion of molecular adjuvants perhaps in combination with targeting carrier systems such as nano- and microparticles. Although transfection levels are often low, studies have demonstrated a persistence of transgene expression at the injection site that might coincide with a time when the fish would normally be ready for slaughter [67]. There have been experiments with suicidal DNA vaccines for fish, where the plasmid vector includes a protein to induce apoptosis after an immune response has been triggered [26].

## 11. PLGA particles as carrier systems for DNA vaccines – focus on fish

Besides the proven efficacy of G-protein expressing plasmids against novirhabdoviruses, there is an urgent need to develop high performing vaccines against other viral diseases. In this aspect, DNA vaccines delivered by several kinds of particles may show promise. There is considerable research being done on the application of carrier systems and/or DNA-complexes to facilitate higher levels of entry and reduce degradation, reviewed by Saade et al. [65]. The potential of PLGA particles as adjuvants and carriers for DNA vaccine delivery has received considerable attention in mammalian studies [68]. In spite of this, reports on the use of PLGA particles for DNA delivery to fish are nearly non-existent. In 2008, Tian et al*.* were the first to report on the use of PLGA microcapsules containing a plasmid vaccine for the oral immunization of Japanese flounder against lymphocystis disease virus (LCDV) [69]. Following immunization they detected transgene expression in gills, intestine, spleen and kidney from fish vaccinated with encapsulated pDNA. The encapsulated pDNA also induced higher levels of antibodies compared to control fish injected with naked pDNA. Tian and Yu later demonstrated a significant increase in resistance to LCDV infection after oral administration of a pDNA vaccine encapsulated into PLGA nanoparticles [70]. Finally, the last study so far reported was by Adomako et al., who used PLGA nanoparticles incorporated into feed for oral delivery of a DNA vaccine against IHNV to rainbow trout [28]. They found that particles were mostly taken up in the posterior gut, but that a significant number of fish that showed uptake in gastrointestinal epithelial cells did not demonstrate detectable levels of transgene expression. Although antibody responses could be detected in fish given high doses of pDNA, the RPS at a six weeks post-vaccination challenge was still only 22%. No VHSV/IHNV DNA vaccine trials using nano- or micro-particle vehicles have been carried out so far. It would have been very interesting to compare the vaccine efficacy of naked plasmid encoding for VHSV and IHNV G-protein to corresponding particle delivered vaccines.

The exploitation of PLGA encapsulated DNA vaccines in fish is new, currently there are no information whether this strategy is a way to go to increase efficacy of DNA vaccines for fish. There are, however, indications that pDNA encapsulated in PLGA nanoparticles induce a antiviral immune response in salmon - at a higher level than what is achieved with only pDNA [71].

## 12. Issues of making DNA encapsulated PLGA particles

The w1/o/w2 method described previously is frequently used for the encapsulation of pDNA into PLGA particles, but results are highly variable with regard to encapsulation efficiency and loading as well as DNA degradation and release. The hydrophilic nature of DNA complicates the process of encapsulation as it increases the risk of plasmid diffusion into the w2 phase during solvent evaporation [72], and the encapsulation process also offers multiple challenges in terms of preservation of bioactivity – i.e. keeping the supercoiled DNA topoform intact.

Among the factors known to affect the integrity of the pDNA to be encapsulated are the polymer composition and molecular weight (Mw), shear force, preparation temperatures, solvents and the concentration and Mw of the applied stabilizer [73]. In general, polymers with a high Mw also result in the highest encapsulation efficiencies and lower the burst release of plasmid DNA [73].

## 13. Transgene expression and immune responses by PLGA-encapsulated pDNA

Encapsulated DNA has been shown to be more potent than naked DNA at mediating transgene expression in vitro in a variety of mammalian cells types [74]. However, in vivo studies report on a superiority of naked DNA in eliciting transgene expression not only compared to PLGA particles, but also to other formulated DNA vaccines as well [74].

Small particles (< 100 nm) are often shown to be internalized more rapidly, and also show the highest transfection efficiencies [75].

The adsorption of plasmid DNA onto the surface of PLGA particles, rather than encapsulation, has been reported to result in a higher transgene expression, but the expression declined more rapidly [48].

In addition to the pro-inflammatory cytokines induced by empty PLGA nano- and micro-particles (IL-1β and TNF-α) [76], the use of different particle sizes might influence the resulting cytokine profile after administration of encapsulated or particle-bound CpG DNA [77]. In addition to pro-inflammatory cytokines, nanoparticles have been shown to also enable an induction of antiviral cytokines such as type I IFNs in addition [77]. The encapsulation of pDNA encoding antigens has also been shown to elicit CTL-responses, even with pDNA-amounts that elicited no such responses after naked administration [77]. Moreover, encapsulated pDNA also enhanced the total antibody response at high doses, while inducing a more rapid and complete sero-conversion when lower doses were applied [78].

The injection of PLG microspheres into muscle has been shown to result in a foreign body response, with a large influx of different inflammatory cells that appear largely related to microspheres especially at later time-points [79]. These infiltrating cells were also the ones that were primarily transfected, an observation similar to that found in another study [80].

## 14. Other particles in vaccine delivery to fish

The extensive use of PLGA as vaccine delivery vehicles can largely be attributed to their high biocompatibility as well as the ease with which the particles can be prepared. There are other means to increase the level of transgene expression that may increase vaccine efficacy, given that the transgenes produced display fair immunogenic properties, by using tailored particle systems – as outlined by Nguyen et al. [73]. Other particles have also been investigated as carrier systems for vaccination of fish (Table 2), either on their own or in combination.

**Table 2:** Compounds tested as adjuvants and gene vaccine delivery systems to fish species, published from 2007 to date

**Particles**	**Carrier molecule(s)**	**Assembly**	**Species**
**Alginate**	ß(1,4)-D-mannuronic and α(1,4)-L-guluronic acid residues	Encapsulation	Japanese flounder [81]
**Chitosan**	ß(1,4)-D-glucosamine and N-acetyl-D-glucosamine copolymer	Encapsulation	Nile tilapia (*Oreochromis niloticus)*[82]
			Japanese flounder [83]
			Asian sea bass (*Lates calcifer*) [84]
			Turbot [85]
**Liposomes**	Artificial lipid bilayer vesicles	Encapsulation	Kelp grouper (*E. bruneus*) [86]
**Polycaprolactone**	Biodegradable, synthetic polymer	Coating	Indian major carp (*Labeo rohito*) [87]
**Calcium phosphate**	Inorganic, biodegradable and biocompatible material	Coating	Indian major carp [88]

## 15. Current challenges in the use of PLGA particles as carriers/adjuvants

Despite a growing number of optimistic reports on the adjuvant/carrier properties of PLGA particles, there are still many hindrances to be overcome. One of these is the preparation method, where different drugs/vaccines require different conditions. While some compounds are easily encapsulated, others – like DNA vaccines – are more difficult to encapsulate in an efficient manner. Low encapsulation efficiencies not only result in low antigen loading, but also mean that a large amount of drug/vaccine goes to waste during particle preparation. The detrimental effects that encapsulation may have on certain antigens such as pDNA are also major limiting factors at the moment, and need to be resolved. Particle preparation is also low-scale work as of yet, with considerable effort to be made before the process is optimized on a large scale suitable for mass-production.

## 16. Concerns regarding PLGA nano- and micro-particles

Any construct/compound, when brought down to sub-micron sizes, will exhibit new and potentially harmful characteristics [89]. The small size means they can interact with biological membranes in an entirely new way, thus inducing responses not seen with larger constructs. As with all new applications it takes time to fully survey the potential side effects. In vitro studies using PLGA nano- and micro-particles have so far not revealed any toxic effects, even at large doses [90]. The various concerns regarding the use of PLGA particles in aquaculture vaccines have recently been reviewed by Nielsen et al. [91].

## 17. Safety and regulatory aspects by DNA vaccines

Safety aspects include potential effects on the vaccinated animals, the environment and the consumer [1,64,92]. Other safety issues include potential shed of the vaccine to the environment from the vaccinated animal and by predatory animals [93]. Human safety does also, although with low probability, include potential effects by self-injection by vaccinators. These safety aspects need to be taken into consideration by relevant authorities when safety aspects are to be documented.

The Canadian Food Inspection Agency (CFIA) that gives authority to the Veterinary Biologics Section (VBS) of the Animal Health and Production Division (AHPD) approved the IHNV DNA vaccine for commercial use in Canada in 2005 [94]. Five aspects were taken into consideration before the vaccine was approved: (i) public perception and acceptance, (ii) regulatory and environmental concerns, (iii) risk-benefit, (iv) feasibility of producing the vaccine at a scale and cost appropriate for the fish industry, and (v) intellectual property issues [91]. At present there has not been approved any DNA vaccine to be used in aquaculture in Europe. To obtain marketing authorization within Europe, a new veterinary medicine has to meet the criteria and requirements of the EU pharmaceutical legislation for both medical and veterinary applications. To achieve a marketing application a MAA (marketing authorization application) has to be submitted to the European Medicines Agency (EMA). The EMA has drafted guidelines for the veterinary use of DNA vaccines [95]. The European guidelines include several aspects to be considered in order to conduct a risk assessment of DNA vaccines. This includes i) the possibility of pDNA integrating into the chromosome, ii) concerns about possible adverse effects on the immune system, iii) risks posed by the additional use of genes encoding cytokines or co-stimulatory molecules or iv) undesirable biological activity by the expressed antigen itself. At present there are work undertaken within EMA with the intention to revise and update the guidance for DNA vaccines and the goal is to provide a specific guidance document [96]. This work is especially concerned to the use of DNA vaccines to humans and if the outcome will have influence on the veterinary use of DNA vaccines is therefore uncertain. The guidelines prepared by the FDA (US) points to some of the same areas as in EU, and recommends that safety testing should include tests on vaccine immunogenicity, effects from cytokines and other immunomodulatory genes, autoimmunity, local reactogenicity and systemic toxicity and studies of bio-distribution, persistence and integration [97].

The issue of plasmid persistence and chromosomal integration of DNA vaccine is of relevance for both safety and policy [92,93]. Norwegian authorities may for example, due to uncertainties with regard to whether DNA vaccines persist degradation in tissues and organs label DNA vaccinated fish as a GMO [98]. If a DNA vaccinated animal is considered to be a GMO the producers has also to meet the requirements of the EU environmental legislation on the deliberate release of GMOs (Directive 2001/18/EC). The objective of an environmental risk assessment (ERA) in accordance with Directive 2001/18/EC is to identify and assess on a case-by-case basis the potential harmful effects of a GMO for humans, animals (domestic and wildlife), plants, microorganisms and the environment. The EMA has developed two specific guidelines for the preparation of ERA to facilitate adoptions of the requirements and the methodology of the Directive to GMO-containing medical products. Although these guidelines have mainly been used for MAA of GM vaccines, they may provide necessary information and procedures that can also be used to perform ERA for DNA vaccines and vaccinated animals.

The process of obtaining a market authorization for a new fish vaccine is a both time consuming and expensive. To overcome some of the legal uncertainties there is a need to assess potential consequences on a case-by-case basis and to be aware of that a number of factors such as the inserted gene, other gene inserts of the pDNA, injection site and amount of vaccine injected are of importance. Safety and regulatory uncertainties are related to distribution and degradation of the DNA after injection and includes that there is a need of research on the (i) the stability of the DNA vaccine, (ii) plasmid persistence, (iii) unintended immunological impacts, and (iv) potential for integration of the pDNA into the chromosome of the recipient organism [93]. These uncertainties are also of relevance for consumer acceptance and the markets confidence in safety.

The GMO issues may only be of concern for countries that have specific GMO legislation and that demands that GMOs and products thereof need to be labeled. In USA and Canada there are no requirements for labeling of food containing GMOs, and they do not have specific GMO legislation. In Europe due to the uncertainties with regard to the persistence of a DNA vaccine, the vaccinated fish may need to be labeled as a GMO. For example European countries that employ restrictions on GMO may not import the DNA vaccinated fish, and if the fish need to be labeled as a GMO this will have an influence on consumer willingness to buy it. Ultimately may these uncertainties prevent the use of DNA vaccines [92].

## 18. Conclusions

There is a crucial need to increase efficacy of DNA vaccines against persistent and hard-to-combat viral infections, this can be met by: (i) Application of vaccine carriers to increase the uptake in antigen presenting cells – followed by enhanced presentation of transgene peptides/antigens, (ii) Use of nano-scale particles to increase the level of cross presentation by such cells – this may also be beneficial to produce antibody response as well as cell mediated immunity, and (iii) Employing additional adjuvants such as TLR ligands, other than RNA and/or DNA, ligands to boost the response considerably. Safety and regulatory uncertainties are related to distribution and degradation of the DNA after injection and more effort needs to be put into gaining understanding of the mechanisms of pDNA uptake, from the moment of administration until the stage of transcription and translation in the nucleus.

## Competing interests

The authors declare that they have no competing interests.

## Authors’ contributions

LBH has made the most substantial contribution to this review, while RAD and AIM has revised the manuscript critically. All authors read and approved the final manuscript.
